# Turning Tissue Waste into High-Performance Microfiber Filters for Oily Wastewater Treatment

**DOI:** 10.3390/ma13020378

**Published:** 2020-01-14

**Authors:** Gaoliang Wei, Jun Dong, Jing Bai, Yongsheng Zhao, Chuanyu Qin

**Affiliations:** Key Laboratory of Groundwater Resources and Environment (Ministry of Education, China), College of New Energy and Environment, Jilin University, Changchun 130021, China; glwei@jlu.edu.cn (G.W.); dongjun@jlu.edu.cn (J.D.); baijing927@jlu.edu.cn (J.B.); qincyu@jlu.edu.cn (C.Q.)

**Keywords:** biopolymer microfiber, oil/water separation, antifouling, filter, oily wastewater

## Abstract

Developing low-cost, durable, and high-performance materials for the separation of water/oil mixtures (free oil/water mixtures and emulsions) is critical to wastewater treatment and resource recovery. However, this currently remains a challenge. In this work, we report a biopolymer microfiber assembly, fabricated from the recovery of tissue waste, as a low-cost and high-performance filter for oily wastewater treatment. The microfiber filters demonstrate superhydrophilicity (water contact angle of 28.8°) and underwater superoleophobicity (oil contact angle of 154.2°), and thus can achieve separation efficiencies of >96% for both free oil/water mixtures and surfactant-stabilized emulsions even in highly acidic (pH 2.2)/alkaline (pH 11.8) conditions. Additionally, the prepared microfiber filters possess a much higher resistance to oil fouling than conventional membranes when filtering emulsions, which is because the large-sized 3D interconnected channels of the filters can delay the formation of a low-porosity oil gel layer on their surface. The filters are expected to practically apply for the oily wastewater treatment and reduce the amount of tissue waste entering the environment.

## 1. Introduction

Oily wastewater, generated from diverse sources ranging from oil leakage, crude oil mining, metal processing to domestic sewage, may cause serious environmental problems [1] and the waste of resources, if not properly treated. Many materials with superwetting properties have therefore widely been investigated aiming at removing oil from water and have demonstrated a lot of advantages, such as low energy consumption, high efficiency and simple operation, compared with conventional separation methods including centrifugation, air flotation, coagulation, and flocculation [2,3,4,5]. These superwetting materials, functioning on the basis of intrinsic immiscibility between water and oil, allow the selective permeation of water or oil to realize their mutual separation. To obtain superhydrophilic/underwater superoleophobic or superhydrophobic/superoleophilic materials, their surfaces are usually specially designed with micro/nanostructures, inspired by the natural creatures (such as lotus leaf and water strider) [6,7,8,9], or grafted with hydrophilic/hydrophobic chemical groups [10], or coated with hydrophilic/hydrophobic nanomaterials (such as graphene, metal oxide/hydroxide and zeolite) [11,12,13,14,15,16]. These artificial superwetting materials are therefore relatively expensive. More importantly, they only serve to separate free oil/water mixtures and are generally invalid for oil/water emulsions (oil droplet size is typically less than 20 µm), especially those stabilized by surfactants. This is because their pore size (tens of micrometers) is usually much larger than oil droplets [17].

Alternatively, hydrophilic membrane-based processes can efficiently separate emulsions through size exclusion with oil droplets rejected on their surface and water penetrating the membrane. Unfortunately, these oil droplets can form a low-porosity compressible gel layer with a hydraulic resistance several orders of magnitude higher than that of original membranes [18]. It would thus significantly reduce the membrane flux and greatly increase the costs associated with increased energy consumption and membrane replacement [19]. To retard membrane fouling, many strategies including surface hydrophilization [20,21,22], zwitterionic coating [23,24], photocatalytic cleaning [25] and electrically enhanced antifouling [18,26], have been explored. However, these processes would increase the membrane fabrication cost and consume additional energy. Therefore, it is still a challenge to develop very cheap and high-performance materials capable of separating both free oil/water mixtures and emulsions.

At present, a lot of facial tissues, napkins, and toilet papers are consumed every day. These used biomass materials composed of mainly microfibers are usually thrown into rubbish bin directly, which results in the waste of resources and poses a threat on ecological environment. In this work, considering their superhydrophilic/underwater superoleophobic property, we report that these biomass wastes can be converted into nearly no-cost microfiber filters with a very facile method for the efficient oily wastewater treatment. In view of the good chemical stability of these microfibers, the filters are expected to still function in highly acidic/alkaline environment. Because of the internal 3D pore structure formed by random stacking of microfibers, they are also expected to possess a strong resistance to oil fouling by delaying the formation of the gel layer during filtration of emulsions. In this work, the fabrication, characterization and performance evaluation of the microfiber filters are presented in detail. Additionally, the effect of pore structure on resistance to fouling is also analyzed.

## 2. Materials and Methods

### 2.1. Chemicals and Materials

Acetone, carbon tetrachloride (CCl_4_), hexane, petroleum ether, dimethyl sulfoxide and 1,2-dichloroethane were provided by Tianjin Fuyu Fine Chemical Co., Ltd., Tianjin, China. Hydrophilic polyvinylidene fluoride (PVDF) membranes with an average pore size of 100 nm were commercially available from Merck Millipore Ltd., Darmstadt, Germany. Sodium dodecyl sulfate (SDS) was purchased from Sinopharm Chemical Reagent Co., Ltd., Shanghai, China. The facial tissues composed of bamboo microfibers (BABO^®^ Brand) and wood microfibers (Mind Act Upon Mind^®^ Brand) after cleaning human sweat and soybean oil were as simulated waste. Soybean oil used in this work was clear and transparent edible oil (Arawana^®^ Brand) composed of 100% fat. Vacuum pump oil (100#, Sevenstars^®^ Brand) was produced in Dalian Petroleum Additive Factory. Diesel oil (0#) was purchased from China Petrochemical Corporation.

### 2.2. Preparation of the Microfiber Filters

To remove grease adhering to the tissues after use, they were immersed in acetone under stirring (200 r/min) for 2 h. Then the tissues were fished out and squeezed with tweezers. After the removal of residual acetone with water, the tissues were dried at 60 °C for 4 h. The preparation process of microfiber filters was depicted in Figure S1. In a typical procedure, 2.0 g used facial tissues (1.0 g yellow tissue made of bamboo fibers and 1.0 g white tissue made of wood fibers) were broken in 500 mL deionized water by a household liquidizer to form a turbid pulp. Immediately, the pulp was filtered on a filter paper with an average pore size of 30–50 μm to form a microfiber cake layer. After being dried at ambient temperature (20 ± 2 °C), the cake layer was peeled off the filter paper, and then compressed at 10 MPa for 5 min by a tablet-pressing machine to obtain the microfiber filter. The thickness of microfiber filters was controlled by the pulp volume filtered.

### 2.3. Characterization of the Microfiber Filters

The morphological structures of the microfiber filters were observed on a field-emission scanning electron microscope (SEM, Hitachi S4800, Hitachi corporation, Tokyo, Japan) at an accelerating voltage of 1.0 or 2.0 KV and a current of 5.0 μA. The hydrophilicity/oleophobicity of microfiber filters were evaluated using a contact angle (CA) tester (Fangrui JCY-2, Fangrui Ltd., Shanghai, China). Surface oxygen content was analyzed by an X-ray photoelectron spectroscopy (XPS, ESCALAB 250XI, Thermo Fisher Scientific Ltd., MA, USA). The oxygen-containing groups were identified using a Fourier transform infrared (FTIR) spectrometer (VERTEX 70, Bruker corporation, Karlsruhe, Germany).

### 2.4. Performance Evaluation of the Microfiber Filters

Free oil/water mixtures were prepared by directly mixing oil and water (*v*/*v*, 1:5) under vigorous stirring condition (200 r/min). The oil-in-water emulsion was prepared according to the method reported previously [18]. Typically, 100 mg soybean oil and 10 mg SDS were added into 1 L deionized water. The mixture was then vigorously stirred at 300 r/min for 4 h and probe-sonicated (270 w) for another 30 min. The as-prepared emulsion was used in 12 h. This could guarantee that emulsion was stable enough during the filtration process, because it could be stable for at least 3 days (Figure S2). 

The separation of free oil/water mixtures by microfiber filters was driven by gravity. The filtration module for such a process was schematically shown in Figure S3a. For emulsion filtration, the microfiber filters were tightly sandwiched between two porous metal plates and assembled into a custom-made module (Figure S3b). The filtration was performed with a dead-end mode and driven by a pump. For comparison, the separation performance of commercial PVDF membranes (pore size of 100 nm) was also evaluated with a cross-flow rate of 0.5 m/s. The fluxes (*J*) of the filter and the membrane under a pressure were calculated using the following equation:

*J* = *V*/(*St*) (1)

where *S* is the effective area of microfiber filter or membrane and *V* is the volume of water penetrating the filter or membrane in a time interval (*t*). 

The oil separation efficiency (*R*) of the microfiber filter and membranes were calculated with the following equation:

*R* = (*C*_f_ − *C*_p_)/*C*_f_ (2)

where, *C*_f_ and *C*_p_ were the oil concentration in feed and filtrate, respectively. 

The concentrations of oil in feed and filtrate were analyzed by an infrared spectrometer oil content analyzer (JC-OIL-8) [18]. Before measurements, 10.0 mL feed or filtrate was firstly mixed with 20.0 mL CCl_4_ in a separating funnel. The mixture was then carefully shaken for three minutes. After the complete separation of two phases, CCl_4_ with extracted oil in the bottom layer was collected and measured. The absorbances at 2930, 2960 and 3030 cm^−1^ were recorded, and the oil content was obtained by calculating the absorbance and the correction coefficient.

To recover the flux of the microfiber filters after fouling, they were unloaded from the module and immersed in dimethyl sulfoxide under stirring (50 r/min). After two hours, the filters were fished out, dried, and compressed again at 10 MPa.

## 3. Results and Discussion

### 3.1. Preparation and Characterization of the Microfiber Filters

Washing tissue waste with organic solvent before the preparation of the microfiber filters is a key procedure, because the grease can significantly decrease the hydrophilicity and increase the oleophobicity of microfibers, which is disadvantageous to the separation of oil from water. The subsequent crush of these tissues in water can obtain a turbid pulp (Figure S4). It can be visually observed the biofibers have a length of approximately 1 to 10 mm. These biofibers can be reassembled by vacuum filtration or traditional paper manufacturing process to form a loose flat-sheet structure. To obtain the microfiber filters, a pressure is therefore necessary. 

**Figure 1:**
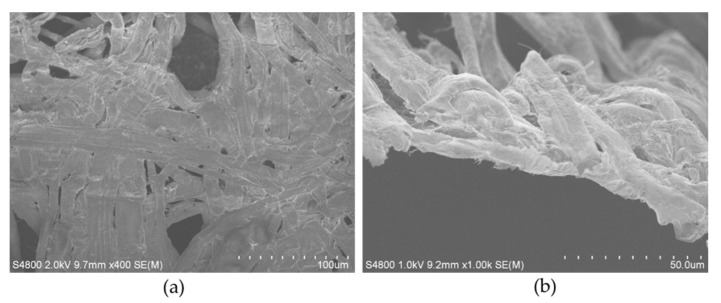
SEM images of a thin microfiber filter: (**a**) surface, (**b**) cross section.

**Figure 2:**
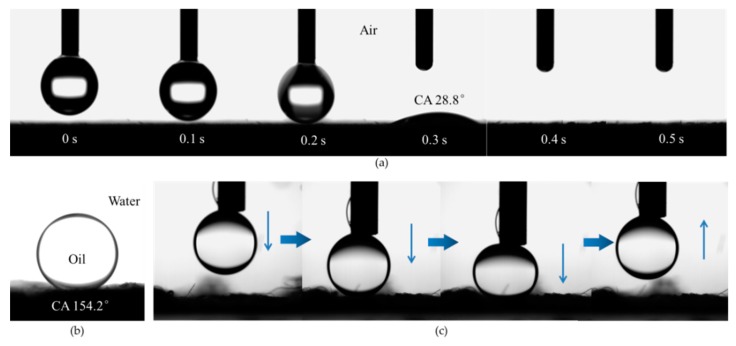
(**a**) Dynamic contact angles of a water droplet on microfiber filter in air; (**b**) Contact angle of 1,2-dichloroethane on microfiber filter in water; (**c**) Photographs of a 1,2-dichloroethane droplet contacting with and leaving from the microfiber filter.

The SEM images of the filters reveal an obvious nonwoven-fabric-like structure, composed of microfibers with diameters of approximately 5–20 μm (Figure 1). It is found that a water droplet can very quickly sink into the microfiber filter within 100 ms, showing an instantaneous contact angle of 28.8° (Figure 2a). This test evidences its excellent hydrophilicity. It is also found that the microfiber filter demonstrates underwater superoleophobicity, which can be confirmed by an oil (1,2-dichloroethane) contact angle of 154.2° in water (Figure 2b). As a result, the 1,2-dichloroethane droplet cannot wet the microfiber filter in water, even under a pressure (Figure 2c). According to the equation Δp = −lγ(cosθa)/A (Supplementary Figure S5a: l is the pore size; γ is the surface tension; θa is the advancing contact angle; A is pore area) [27]), because θa is > 90°, the Δp is > 0, which means the microfiber filter can support a certain pressure. This is the reason that the oil droplet cannot sink into the microfiber filter in water. It is consequently inferred that, with an increase in inclination, the component (F) of gravity of an oil droplet will be larger than adhesion force, then it can theoretically slide down the microfiber filter (Supplementary Figure S5b). As shown in Supplementary Figure S6, a relatively small rolling angle of approximately13° can be experimentally observed, suggesting that the microfiber filter has a weak adhesion force to oil. These interfacial properties, combined with their porous structure, endow the microfiber assemblies with great potential as filters for the treatment of oily wastewater.

**Figure 3:**
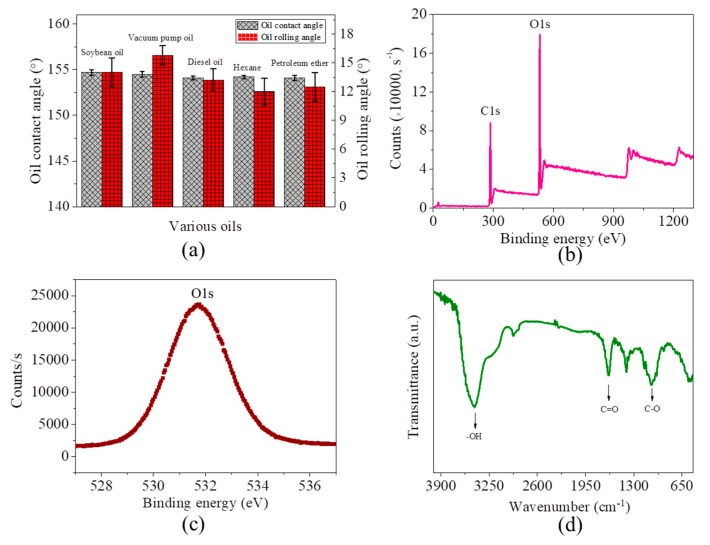
(**a**) Contact angles and rolling angles of five kinds of oils on the microfiber filter in water; (**b**) Oxygen content analysis of the microfiber filters by XPS; (**c**) Symmetric O1s peak centered at 531.6 eV; (**d**) Identification of chemical groups on the microfiber filters by FTIR.

The microfiber filter also demonstrates underwater superoleophobicity towards other oils (for example, soybean oil, vacuum pump oil, diesel oil, hexane and petroleum ether), with all oil contact angles larger than 154° and rolling angles lower than 16° (Figure 3a). To understand the reason for superhydrophilicity/underwater superoleophobicity, the surface chemistry of microfiber filters is consequently investigated. XPS analysis indicates that these microfibers have substantial oxygen element, with an O/C ratio of as high as 0.62 (Figure 3b). The peak O1s centered at 531.6 eV referring to hydroxyl (-OH) shows a symmetric shape (Figure 3c), suggesting that -OH is the main oxygen-containing group. Further analysis by FTIR indicates that, in addition to the -OH, the oxygen atoms can also exist in the form of C=O and C-O (Figure 3d). It thus can be concluded that the excellent hydrophilicity and underwater oleophobicity of the microfiber filters are attributed to the presence of the hydrophilic oxygen-containing functional groups.

### 3.2. Separation of Free Oil/Water Mixtures by the Microfiber Filters

**Figure 4:**
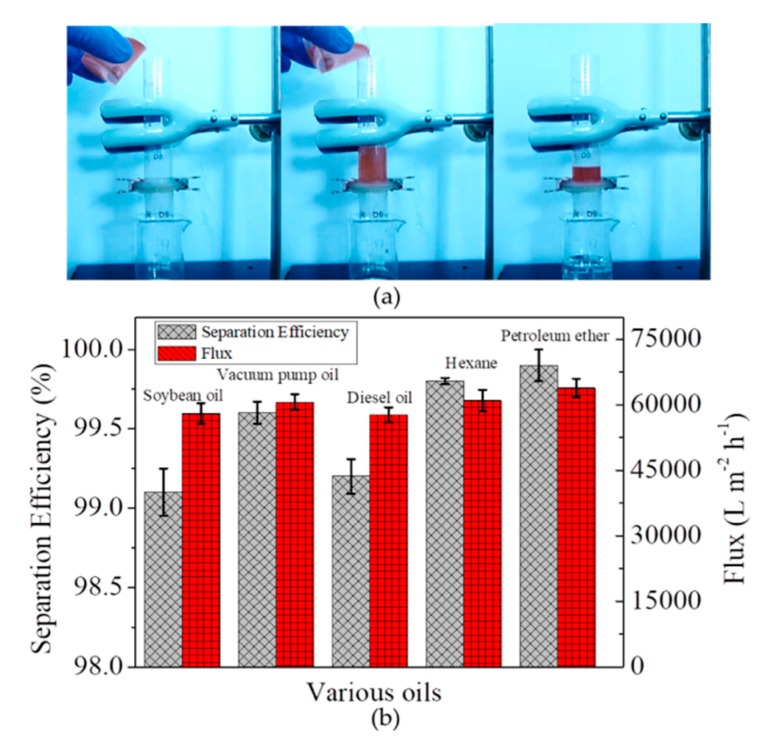
(**a**) Photos showing the separation process of hexane/water mixtures; (**b**) Separation efficiencies of microfiber filters towards five kinds of oils and corresponding fluxes at an altitude difference of 8 cm.

The separation of free oil/water mixtures by the microfiber filter was firstly evaluated, with gravity as a driving force. As shown in Figure 4a, after pouring a mixture of hexane (dyed by Sudan III) and water into the device, the hexane is rejected on microfiber filter while water penetrate it, achieving the separation of hexane and water. The separation efficiencies for oils tested in this work are all higher than 99%, accompanied by a flux of as high as approximately 60,000 L m^−2^ h^−1^ at an altitude difference of 8 cm (Figure 4b). The high separation efficiency and high flux enable the microfiber filters to be high-performance material for water/oil separation.

**Figure 5:**
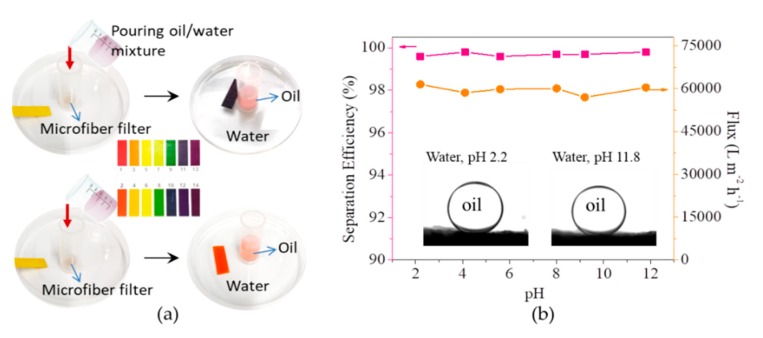
(**a**) Filtration of strong acid/base-based water/oil mixtures; (**b**) Separation efficiencies and fluxes of microfiber filters as a function of pH of water/oil mixtures (Inset shows the oil (1,2-dichloroethane) contact angles on microfiber filters in water at pH 2.2 and pH 11.8).

The biomass microfiber filters can still function in harsh environment such as strong aicd (pH 2.2) and strong base (pH 11.8) condition, which is advantageous over those metal oxide or metal hydroxide-based filters [13,14,28,29,30]. As shown in the inset of Figure 5b, the oil (1,2-dichloroethane) contact angle in water at pH 2.2 is higher than 153.8°, indicating that the microfiber filters can still retain their underwater superoleophobicity at low pH. As a result, they can still separate strong acid-based water/oil mixtures, with oil rejected on their surface and water penetrating them (Figure 5a). Similarly, for strong base-based water/oil mixtures, the microfiber filters can still achieve high separation efficiency. Quantitative measurements show that, at pH ranging from 2.2 to 11.8, the separation efficiencies are all higher than 99.5%, and the fluxes are all approximately 60,000 L m^−2^ h^−1^ at an altitude difference of 8 cm (Figure 5b). Both the separation efficiency and flux are similar with those at the neutral condition. These results indicate the microfiber filters can be chemically stable in the harsh environment, and thus applicable for the separation of oil/water mixtures generated from a variety of activities.

**Figure 6:**
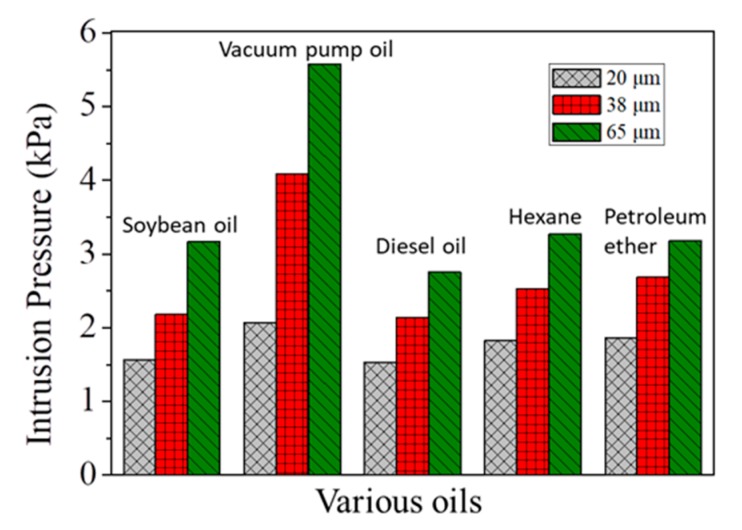
Intrusion pressures of microfiber filters with different thicknesses for various oils.

Apart from separation efficiency and flux, the intrusion pressures of microfiber filters for various oils are also investigated, since it is a very important parameter influencing separation performance. As shown in Figure 6, the intrusion pressures for oils tested are all higher than 1.5 kPa, which suggests the microfiber filters can be applicable in practical separation processes. It is also found that, the intrusion pressure increases with an increase in thickness of microfiber filters for all oils. For example, if the thickness increases from 20 to 65 μm, the intrusion pressure for vacuum pump oil correspondingly increases from 2.1 to 5.6 kPa. This is because, with the increase of thickness, the apparent pore size of the microfiber filter contrarily decreases.

### 3.3. Separation of Emulsion by the Microfiber Filters

Considering the negative correlation between the apparent pore size and the thickness of microfiber filters, we further increase their thickness to aim at the separation of oil-in-water emulsions. However, it is found that the microfiber filter will swell in water, which can enlarge the space between microfibers (i.e., apparent pore size of the filters). As a result, the filters still cannot reject oil micro-droplets in water, even their thickness is increased to 1.0 mm (this value is measured on dry condition). Therefore, we sandwich the microfiber filter between two porous metal plates before it contacts with water (schematically shown in Figures S3b and S7a). Analysis result shows that the oil concentration in filtrate is as low as 3.8 mg L^−1^, which is lower than the maximum allowable discharge concentration of 10 mg L^−1^ [18] and indicates an oil removal rate of approximately 96%. Direct visual observation also confirms the good separation efficiency, as there are many oil drops in feed, but not in filtrate (Supplementary Figure S7b). 

**Figure 7:**
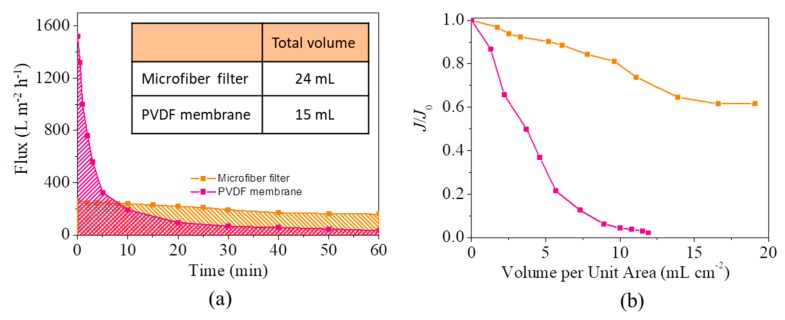
(**a**) Fluxes of microfiber filter and PVDF membrane decline with time (Effective area: 3.2 cm^2^; Pressure difference: 0.4 bar). Inset shows the total volume of emulsion treated by microfiber filter and PVDF membrane in one hour; (**b**) Flux retention rate (*J*/*J*_0_) as a function of filtrate volume per unit area (mL cm^−2^).

It is surprised to find that the microfiber filters possess a quite higher resistance to oil fouling than conventional hydrophilic PVDF ultrafiltration membrane (Supplementary Video S1). As shown in Figure 7a, during a one-hour test at 0.4 bar, the flux of the microfiber filter slowly declines from original 260 L m^−2^ h^−1^ to 160 L m^−2^ h^−1^. By contrast, the flux of PVDF membrane shows a sharp decline and is the same with that of microfiber filter in the eighth minute. After an operation of 1 h, the membrane flux is 30 L m^−2^ h^−1^, only 2% of the original value. Consequently, despite having a lower flux than PVDF membranes, the microfiber filters can treat more wastewater than them within one hour (inset of Figure 7a). To further illustrate the high resistance of microfiber filters to fouling, the flux retention rate (J/J_0_, a ratio of the flux after fouling to original flux) as a function of filtrate volume per unit area (mL cm^−2^) is established. As shown in Figure 7b, after the same volume of emulsion is treated by the microfiber filter and PVDF membrane with the same effective area, the flux retention rate of microfiber filter is obviously higher than that of PVDF membrane. Specifically, after a treat quantity of 19.1 mL cm^−2^, the microfiber filter still has a flux of 61.5% of original value (J/J_0_ = 65%). This flux retention rate is much higher than that of PVDF membrane (2.2%), even though the membrane has a lower treat quantity of 11.9 mL cm^−2^. It should be admitted that the PVDF membranes have a higher oil removal (>99%) than the microfiber filter (~96%), because the membranes have a much smaller pore size (100 nm).

**Figure 8:**
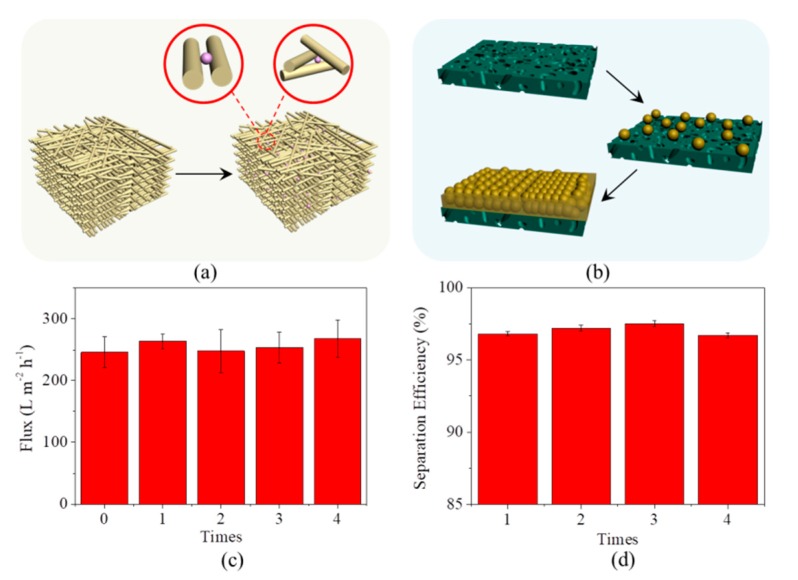
(**a**) Scheme for the illustration of the location of oil drops after being rejected by microfiber filter; (**b**) Scheme for the illustration of the location of oil drops after being rejected by PVDF membrane; (**c**) Fluxes of microfiber filter as a function of clean times; (**d**) Separation efficiencies of microfiber filter as a function of clean times.

The high resistance of microfiber filters to oil fouling can be attributed to their internal 3D pore structure formed by the stacking of microfibers, relatively large pore size, and thick structure. These structural characteristics allow oil drops to be rejected by both their surface pores and internal pore channels (Figure 8a), which can delay the formation of oil gel layer with a quite low porosity and a hydraulic resistance 2–3 orders of magnitude higher than those of solid particles or undistorted spheres on the surface [31,32]. For comparison, the small pore size (100 nm) of PVDF membranes enables the rejection only by their surface pores, which will results in the formation of compressible oil gel layer and finally serious fouling (Figure 8b). If their pore size is increased to be the same as that of microfiber filters, the oil separation efficiency of PVDF membranes will significantly decline because their thin structure (approximately 120 μm) cannot provide adequate internal sites for oil rejection. Such an analysis suggests that antifouling is an intrinsic advantage of the microfiber filters over separation membranes, rather than a result obtained by deliberately choosing specific parameters. 

Additionally, the flux of the microfiber filters can be easily recovered after fouling, just by soaking them in dimethyl sulfoxide for two hours and subsequently compressing them. An immersion in dimethyl sulfoxide is aimed at the removal of oil trapped by the filter. This process will not damage the bio-microfibers, thanks to their chemical stability towards most of organic solvents. To reduce the pore size of the microfiber filter, they therefore need to be compressed again after washing. The two simple steps can allow an almost 100% flux recovery, which has been evidenced by the experimental result that all fluxes after every clean (totally four times) are not lower than original one (Figure 8c). At the same time, the microfiber filters can still maintain their high separation efficiency (>96%, Figure 8d). It should be emphasized that many other organic solvents, for example, petroleum ether and ethyl acetate, can be also competent for their regeneration. 

## 4. Conclusions

The microfiber assemblies fabricated from the recovery of tissue waste can be utilized as high-efficiency filters for the separation of water/oil mixtures (even emulsion). The chemical stability of the microfiber filters allows for efficient separation in highly acidic/alkaline conditions. When applied for the treatment of emulsions, the microfiber filters possess a quite high resistance to oil fouling by delaying the formation of a gel layer, benefiting from their structural characteristics (internal 3D pore structure, relatively large pore size, and thick structure). Additionally, the microfiber filters can be easily regenerated after fouling, just by immersing them in dimethyl sulfoxide for two hours and subsequently compressing them. This work demonstrates a worthwhile example for waste reuse and is thus expected to be competent in the treatment of oily wastewater, in consideration of the excellent performance of the almost no-cost microfiber filters.

## Supplementary Materials

The following are available online at https://www.mdpi.com/1996-1944/13/2/378/s1, Figure S1: Preparation process of microfiber filters derived from tissue waste, Figure S2: Diameter distribution of oil droplets in as-prepared emulsion and the one after standing for 3 days, Figure S3: Schematic illustration of the setups for filtration of free oil/water mixtures (a) and emulsion (b), Figure S4: (a) Tissue before crush in water; (b) Obtained turbid pulp, Figure S5: (a) Schematic illustration of wetting modes of the oil/water/microfiber system; (b) Schematic illustration of the change of force exerted on an oil droplet with the increase of inclination angle, Figure S6: Rolling angle measurement of 1,2-dichloroethane on filter surface, Figure S7: (a) Illustration of the sandwich-like structure of the microfiber filter and two porous metal plates; (b) Optical microscope images of emulsion before and after filtration, Video S1: Flux decline with the increase of time.
