# Supplementary material for: Turning Tissue Waste into High-Performance Microfiber Filters for Oily Wastewater Treatment
Source: Materials (Basel). 2020 Jan 14;13(2):378. doi: 10.3390/ma13020378 (PMC7013821; doi:10.3390/ma13020378)
Supplement: Supplementary file 1 [file materials-13-00378-s001.pdf]

materials

Supplementary Materials

Turning Tissue Waste into High-Performance Microfiber Filters for Oily Wastewater Treatment

Gaoliang Wei, Jun Dong, Jing Bai, Yongsheng Zhao * and Chuanyu Qin

Key Laboratory of Groundwater Resources and Environment (Ministry of Education, China), College of New Energy and Environment, Jilin University, Changchun 130021, China; glwei@jlu.edu.cn (G.W.); dongjun@jlu.edu.cn (J.D.); baijing927@jlu.edu.cn (J.B.); qincyu@jlu.edu.cn (C.Q.)

* Correspondence: zhaoyongsheng@jlu.edu.cn; Tel.: +86-413-8502608

Received: 4 November 2019; Accepted: 31 December 2019; Published: date

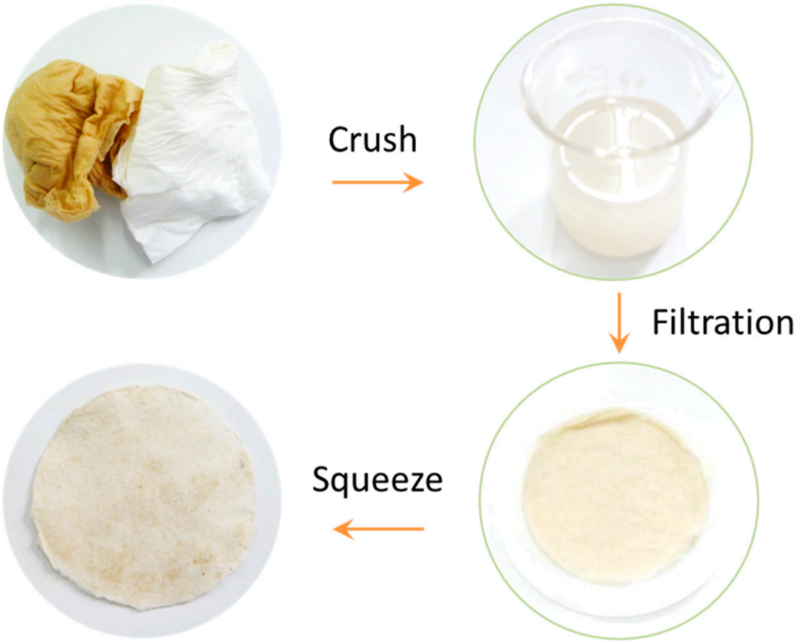

Figure S1. Preparation process of microfiber filters derived from tissue waste.

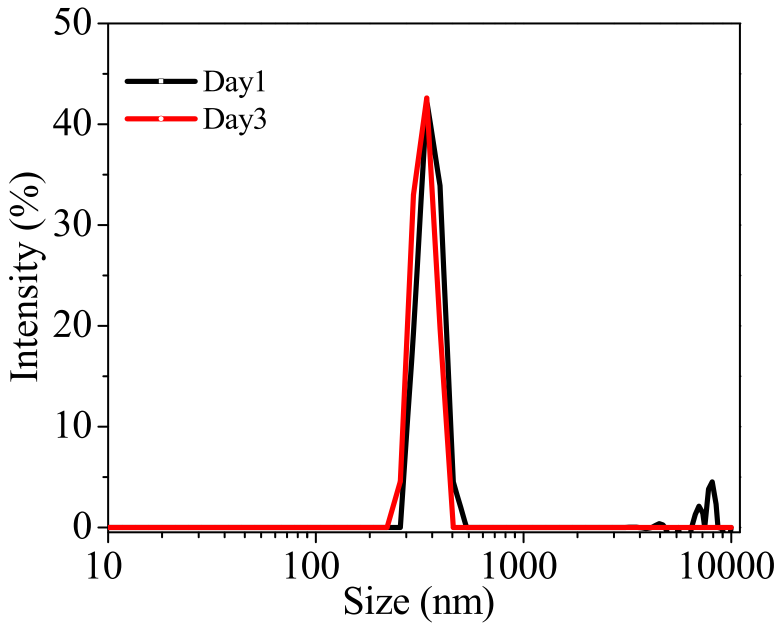

Figure S2. Diameter distribution of oil droplets in as-prepared emulsion and the one after standing for 3 days. The diameter distribution of oil droplets do not change with the increase of time within 3 day, which suggests the emulsion can be stable for at least 3 day.

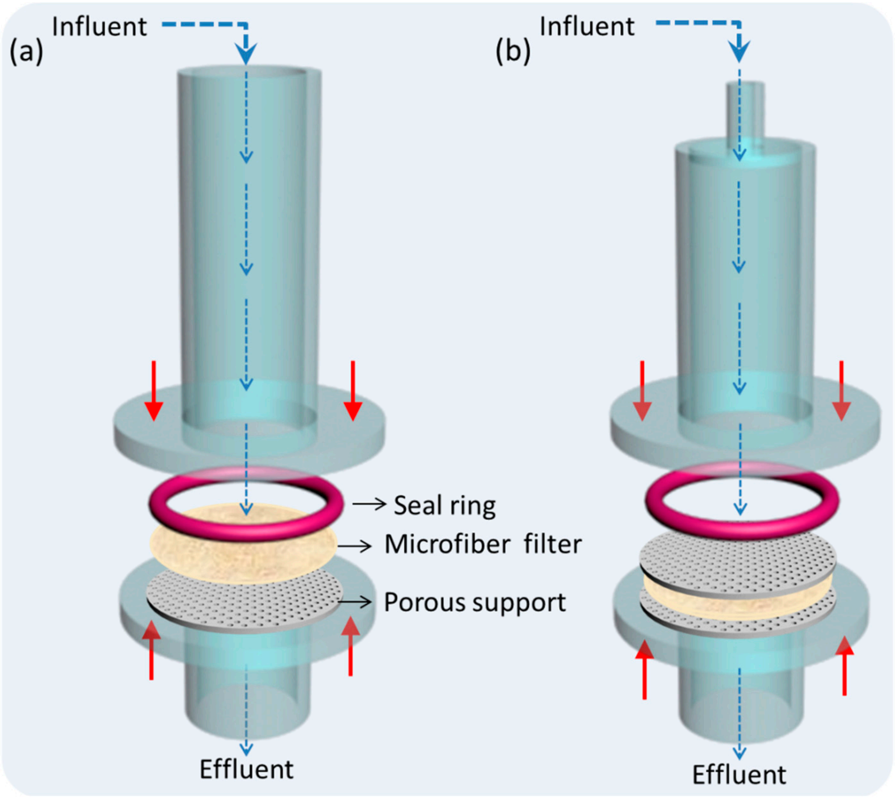

Figure S3. Schematic illustration of the setups for filtration of free oil/water mixtures (**a**) and emulsions (**b**).

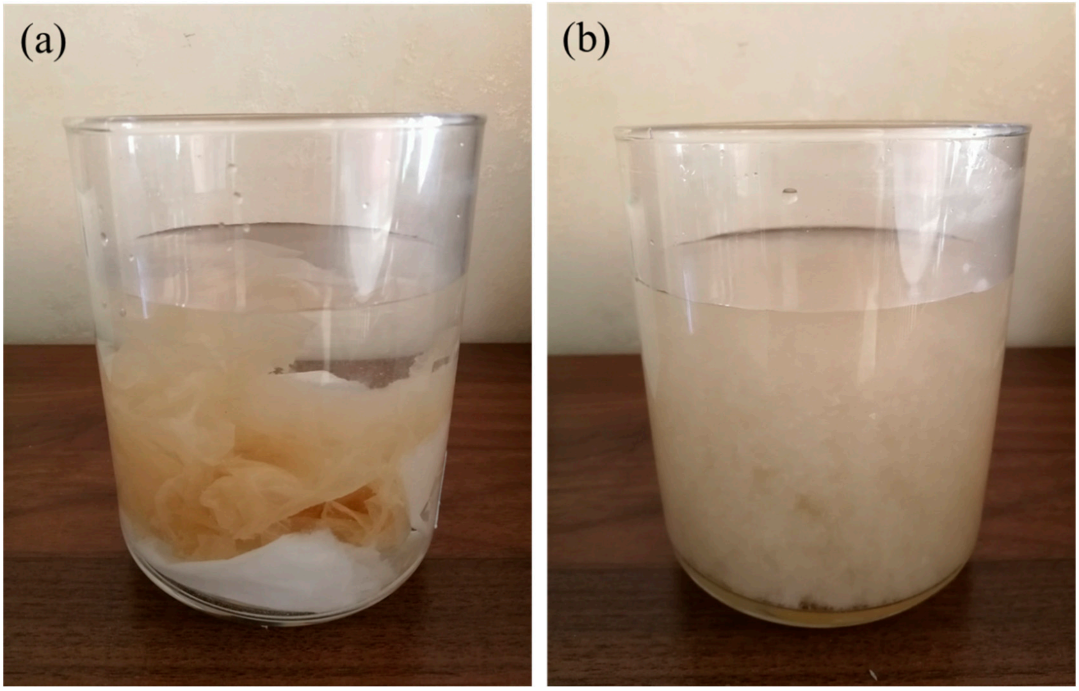

Figure S4. (**a**) Tissue before crush in water. (**b**) Obtained turbid pulp.

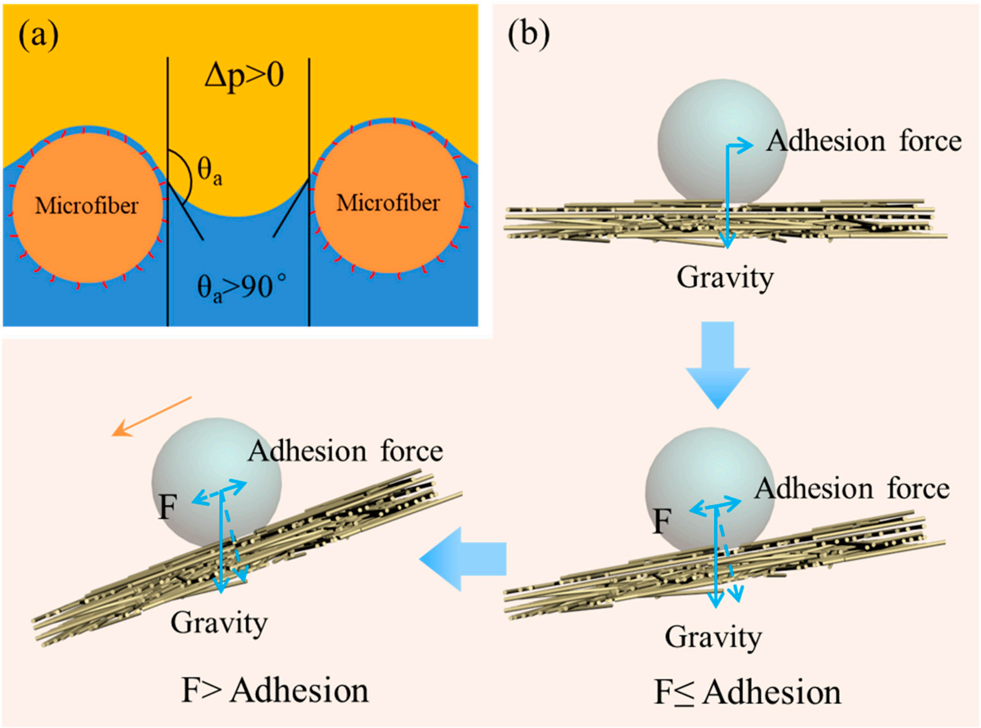

Figure S5. (**a**) Schematic illustration of wetting modes of the oil/water/microfiber system. (**b**) Schematic illustration of the change of force exerted on an oil droplet with the increase of inclination angle.

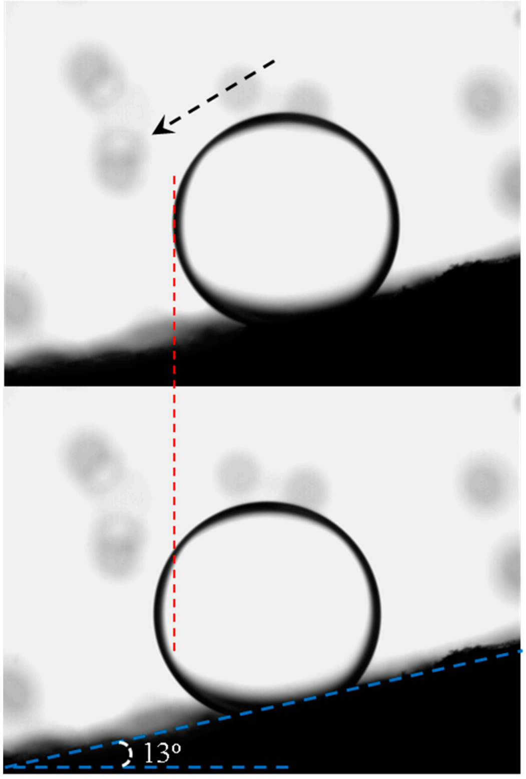

Figure S6. Rolling angle measurement of 1,2-dichloroethane on filter surface.

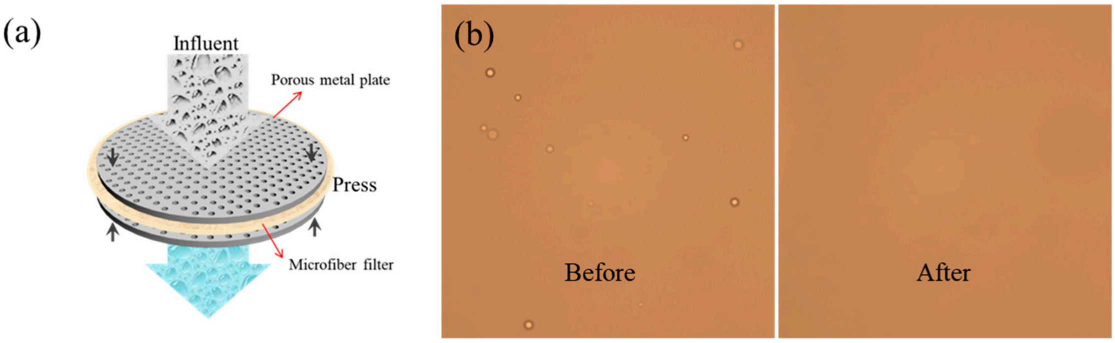

Figure S7. (**a**) Illustration of the sandwich-like structure of the microfiber filter and two porous metal plates. (**b**) Optical microscope images of emulsion before and after filtration.

© 2019 by the authors. Submitted for possible open access publication under the terms and conditions of the Creative Commons Attribution (CC BY) license (http://creativecommons.org/licenses/by/4.0/).
